# Effective Connectivity in Spinal Cord Injury-Induced Neuropathic Pain

**DOI:** 10.3390/s22176337

**Published:** 2022-08-23

**Authors:** Radha Kumari, Mohammed Jarjees, Ioana Susnoschi-Luca, Mariel Purcell, Aleksandra Vučković

**Affiliations:** 1 Biomedical Engineering Research Division, University of Glasgow, Glasgow G12 8QQ, UK; 2 Medical Instrumentation Techniques Engineering Department, Northern Technical University, Mosul 41002, Iraq; 3 Queen Elizabeth National Spinal Injuries Unit, Queen Elizabeth University Hospital, Glasgow G51 4TF, UK

**Keywords:** spinal cord injury, central neuropathic pain, motor imagery, EEG, source reconstruction, effective connectivity

## Abstract

Aim: The aim of this study was to differentiate the effects of spinal cord injury (SCI) and central neuropathic pain (CNP) on effective connectivity during motor imagery of legs, where CNP is typically experienced. Methods: Multichannel EEG was recorded during motor imagery of the legs in 3 groups of people: able-bodied (*N* = 10), SCI with existing CNP (*N* = 10), and SCI with no CNP (*N* = 20). The last group was followed up for 6 months to check for the onset of CNP. Source reconstruction was performed to obtain cortical activity in 17 areas spanning sensorimotor regions and pain matrix. Effective connectivity was calculated using the directed transfer function in 4 frequency bands and compared between groups. Results: A total of 50% of the SCI group with no CNP developed CNP later. Statistically significant differences in effective connectivity were found between all groups. The differences between groups were not dependent on the frequency band. Outflows from the supplementary motor area were greater for the able-bodied group while the outflows from the secondary somatosensory cortex were greater for the SCI groups. The group with existing CNP showed the least differences from the able-bodied group, appearing to reverse the effects of SCI. The connectivities involving the pain matrix were different between able-bodied and SCI groups irrespective of CNP status, indicating their involvement in motor networks generally. Significance: The study findings might help guide therapeutic interventions targeted at the brain for CNP alleviation as well as motor recovery post SCI.

## 1. Introduction

Spinal cord injury (SCI) is a devastating neurological condition that results in physical disability and other secondary consequences such as central neuropathic pain (CNP) [1]. CNP is present in approximately 65% of people with SCI, in which the person experiences pain, typically described as burning, tingling, stabbing, shooting, or an aching sensation at or below the level of injury. CNP can happen even in complete injuries where there is no motor or sensory function preserved below the level of injury [1]. 

Neurorehabilitation interventions that promote recovery and discourage maladaptive neuroplasticity, manifesting as CNP or other conditions, include transcranial direct current stimulation [2], neurofeedback [3], and brain-computer interfaces [4]. To this end, brain reorganization post SCI can be studied using neuroimaging modalities such as functional magnetic resonance imaging (fMRI) and electroencephalography (EEG) to guide brain-targeted interventions. These studies record activity in a resting state or during movement tasks, analyse the corresponding activation or connectivity and often compare it with an able-bodied control group. Movement tasks typically include motor imagery (MI) or motor attempts. The former involves the person imagining the limb moving without actually moving it, whereas the latter is relevant for people with paralysis who attempt the movement but are not able to complete the execution. Most studies either exclusively study SCI-induced CNP or do not control for the presence of CNP in SCI, as it is inevitably present in the majority of people with SCI.

There have been many studies demonstrating neuroplasticity through changes in brain activity post-SCI during the resting state [5,6,7,8]. In the subacute phase, people with SCI show reduced alpha activity compared to able-bodied controls [5]. In the chronic phase also, people with SCI show reduced alpha activity along with increased theta and beta activity [6]. Further, in the chronic phase, compared to people with SCI and no CNP, people with SCI and CNP show a significant slowing of the EEG spectrum [7,8]. Several studies have shown brain reorganization corresponding to movement and reported changes in brain activation compared to able-bodied people, both in terms of intensity and areas involved [9,10,11,12,13]. Particularly, in subacute SCI, posterior shifts in activation during MI or motor attempts of both affected and unaffected limbs have been found, accompanied by increased sensorimotor activation [14]. In the chronic phase, the activations may move back to anterior locations, typically accompanied by motor recovery [14]. In chronic SCI with CNP, brain circuits involved in pain processing such as the prefrontal cortex, anterior cingulate cortex, and insula are also activated [13,15,16]. 

There have also been studies on functional connectivity during a resting state using fMRI and effective connectivity during MI using EEG [17]. The former is inferred from correlations among measurements of neuronal activity and is defined as the statistical dependencies of remote neurophysiological events [18]. The latter refers to the influence of one neural system over another, is dynamic, and depends on a model of interactions or coupling [18]. Both increases and decreases in connectivity between sensorimotor areas have been reported in SCI compared to able-bodied volunteers using resting-state fMRI in both subacute and chronic phases of injury [19,20,21,22]. Studies using EEG and specific MI paradigms have shed further light on effective connectivity in different frequency bands, albeit in chronic SCI [23,24,25,26]. These highlight differential interaction patterns of supplementary motor area and cingulate motor area in SCI and able-bodied cohorts [24,26,27].

The findings on activation patterns and connectivity provide insights into various forms of reorganization post-SCI. However, most of the studies include people with chronic SCI and chronic CNP, and most do not stratify the participants according to the presence of CNP. Furthermore, according to a review on neuroplasticity after SCI and CNP, there still remains limited evidence of structural and functional connectivity changes specific to CNP [28]. We aim to address this research gap, especially in the subacute phase (up to 10 months post SCI [29]) of SCI where early intervention for CNP may be most effective. We used EEG-based source reconstruction to derive effective connectivity between various sensorimotor and pain regions during MI of the legs, an area where CNP is typically present. We compared connectivity between able-bodied and subacute SCI groups at different stages of CNP. The first objective is to differentiate the effects of SCI from the effects of CNP. The second objective is to find predictors of CNP in connectivity patterns. These findings might be relevant for the design of therapies targeting the brain of a person with SCI.

## 2. Materials and Method

**Figure 1:**
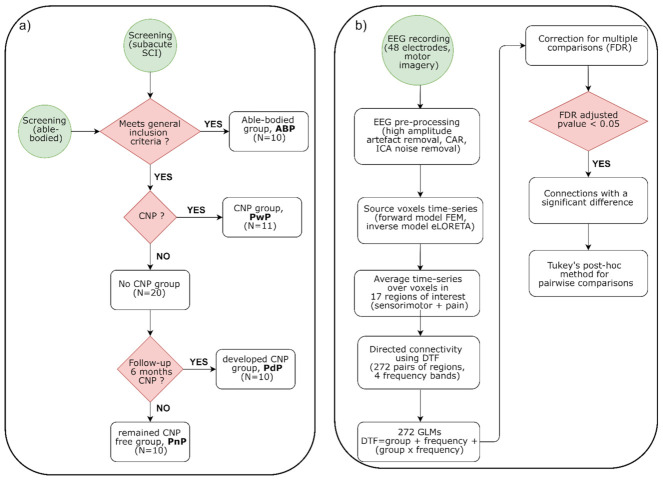
Flow diagram summarizing the methods used in this paper. (**a**) Recruitment and follow-up and (**b**) EEG signal processing and analysis. CAR: common average reference, ICA: independent components analysis, FEM: finite element method, eLORETA: exact low-resolution electromagnetic tomography, DTF: directed transfer function, GLM: generalised linear model, FDR: false discovery rate.

This section has been summarized in the flowchart shown in Figure 1.

### 2.1. Participants

The study is a registered clinical trial (clinicaltrials.gov identifier NCT021789917, accessed on 9 June 2022). People with subacute SCI and satisfying the general inclusion criteria of age 18–75 years, ability to understand the task, and absence of other major neurologic disorder or injury (i.e., stroke, brain injury, epilepsy, multiple sclerosis, cerebral palsy, etc.) were screened for the study. Among the participants screened, the first 11 participants with CNP were recruited. The presence of CNP was confirmed following the criteria by Finnerup et al. [30]. In total, 20 participants with SCI and no CNP were further recruited based on the published literature showing that half of the patients with SCI eventually develop pain within the first year of SCI [31,32]. Then, 10 able-bodied volunteers were recruited for the study with the same general inclusion criteria. Therefore, three groups were formed initially: 10 able-bodied people, 11 people with sub-acute SCI and CNP, and 20 people with subacute SCI and no CNP.

The group with no CNP was followed up for 6 months post-recording to check for CNP. Based on the presence of CNP, they were further divided into 2 groups—people who did not develop pain and people who developed pain. To facilitate the identification of groups, four acronyms are defined—ABP: Able-bodied participants, PwP: SCI participants who had CNP at the time of recording, PnP: SCI participants who did not develop CNP, and PdP: SCI participants who developed CNP.

The emergence of CNP which is a chronic pain was confirmed from patient records as all patients leaving the hospital are required to have regular appointments at 1, 3, and 6 months after discharge. Our spinal unit (Queen Elizabeth National Spinal injuries unit) is the only such hospital in the area, covering a population of 5 million, and people with SCI leaving the hospital remain its lifelong patients, which facilitates follow up. Ideally, any chronic pain should last for 6 months in order to be confirmed as chronic; neuropathic pain can be detected much earlier (sometimes just weeks after injury) due to its characteristic location below the level of injury and characteristic sensory descriptors, such as stinging and burning and shooting pain, often accompanied with allodynia or hyperalgesia (though not often in ASIA A patients, due to the lack of sensation) [33]. 

### 2.2. EEG Data

The EEG data were recorded using three gUSBAmp amplifiers (g.tec medical engineering GmbH, Graz, Austria) using a subset of 48 electrodes [34] from the 10–10 standard EEG electrode recording system [35]. An ear-linked reference was used with AFz acting as ground. The sampling rate was 256 Hz, and the EEG was filtered in real time between 0.5 and 60 Hz with a notch filter at 50 Hz using 5th order IIR Butterworth filters. The electrode impedances were kept below 5 kΩ. 

**Figure 2:**
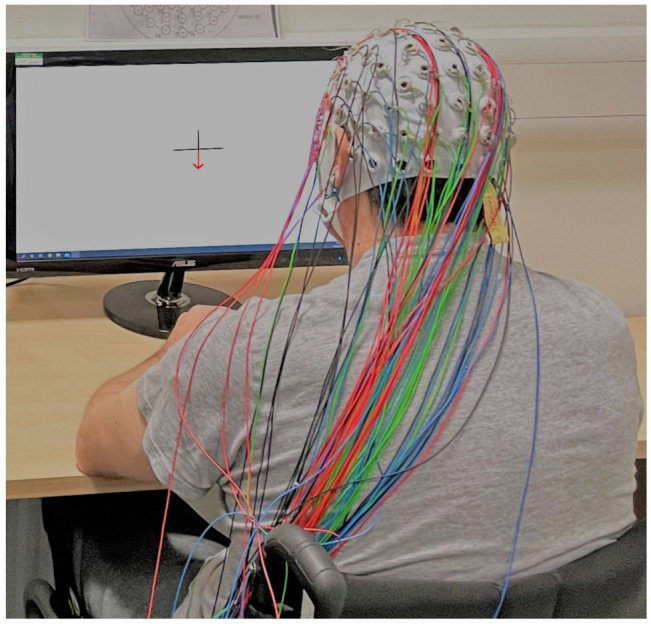
Setup for EEG recording. The downward arrow is the cue to perform motor imagery of the legs. The picture was taken with written consent from the participant.

The recording setup Is shown in Figure 2. Participants were seated approximately 1.5 m in front of a computer screen. A warning cue (cross) was displayed on the screen from *t* = −1 s to *t* = 3 s. At *t* = 0 s, the initiation cue, in the form of an arrow pointing either to the left, right, or down appeared on the screen. The left, right, and downwards arrows represented the left-hand, right-hand, and leg MI, respectively. The initiation cue was displayed until *t* = 1.25 s but the participants had been instructed to continue imagining until the cross disappeared, i.e., for 3 s. The resting period between trials varied between 3 and 5 s to avoid the expectation of a stimulus. There were 6 runs of 30 trials, each lasting 5 min, resulting in a total of 180 trials, 60 for each type of MI.

The EEG signals were preprocessed in EEGLAB [36]. First, the noisy trials with an amplitude above 100 µV were removed. The signals were then re-referenced to a common average reference. Independent component analysis [37], implemented using the Infomax algorithm, was used to remove noisy components representing eye blinks, muscle artifacts, and channel noise. A default residual variance of 0.8 was used to identify noisy components for reference, but the spatial power distribution, power spectrum, and component time series of each independent component for each participant were nevertheless visually inspected alongsidedipole location, to finally remove a noisy component. On average, 3 out of 60 trials were removed per MI type.

### 2.3. Source Time-Series

Time-domain source localisation was performed in the Fieldtrip toolbox to obtain a time series for leg MI trials [38]. This study used only leg MI trials specifically to study CNP because all participants with pain experienced pain in their legs, among other locations, and had sensory-motor functions affected. The forward model was computed using a finite element volume conduction model [39], based on a standard MRI template [40]. The template was segmented into grey matter, white matter, scalp, skull, and cerebrospinal fluid with conductivity values of 0.33, 0.14, 0.43, 0.01, and 1.79 S/m, respectively [41]. The segmented brain volume was divided into a 3D mesh comprised of 4050 hexahedrons (voxels), each belonging to one of the 5 tissues with a resolution of 1 cm.

An inverse model was implemented using “exact low-resolution electromagnetic tomography” (eLORETA) [42]. eLORETA belongs to the family of inverse solutions called LORETA, which calculate current distribution throughout the brain volume, with spatial smoothness as a constraint. LORETA suffers from low spatial resolution. eLORETA attempts to reduce the localisation error of LORETA and gives more importance to the deeper sources. eLORETA is unbiased in the presence of measurement and structural biological noise [43]. The regularization parameter lambda was set to 0.05. The source time series were obtained for 5 s (2 s pre and 3 s post MI cue) in the direction orthogonal to the cortex for each voxel [24].

**Figure 3:**
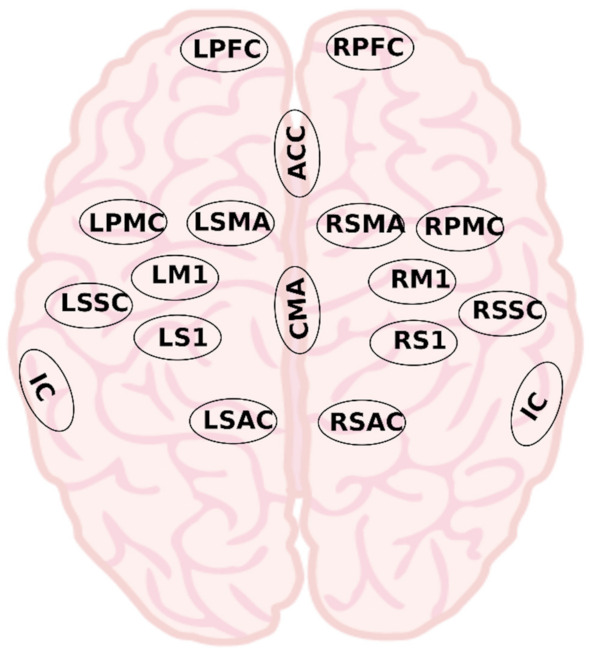
The approximate locations of regions of interest used in this study: right and left premotor cortex (RPMC, LPMC), right and left supplementary motor area (RSMA, LSMA), right and left primary motor cortex (RM1, LM1), right and left somatosensory association cortex (RSAC, LSAC), right and left secondary somatosensory cortex (RSSC, LSSC), right and left primary somatosensory cortex (RS1, LS1), cingulate motor area (CMA), right and left prefrontal cortex (RPFC, LPFC), insular cortex (IC), and anterior cingulate cortex (ACC). The IC is located deep within the lateral sulcus of the brain.

The source time series was then averaged over voxels in 17 regions of interest, spanning sensorimotor and pain areas of the cortex, as shown in Figure 3 [44]. The leg primary motor cortex (M1) and primary sensory cortex (S1) areas were not considered specifically, rather the entire M1 and S1 were included. There were several reasons for this choice. Firstly, the M1 region of legs is located deeper in the central sulcus which limits spatial precision. Previous studies in the field such as Gustin et al. found activation of the M1 leg area during a leg motor imagery task [12]. However, those are fMRI studies which have a high spatial resolution, whereas this is an EEG study with lower spatial resolution. To get reasonable resolution, a source reconstruction method was used, which can be more accurate if subject-specific MRI is used to create a forward model. Since a standard MRI was used, we could not be sure that the same voxel locations would represent leg area in all participants. Secondly, in a study with chronic SCI participants, we showed that the presence of CNP affects sensorimotor cortex globally, thus, to increase the sensitivity of the analysis, the general M1 was considered [13]. Thirdly, movement activation patterns change post SCI as the brain reorganizes, such as lateral and medial shifts in activation [14]. 

### 2.4. Source Connectivity

The source time series were analysed for the period 0.4 s to 1.4 s following the presentation of the MI cue i.e., the motor preparation period. Source connectivity was obtained by fitting a multivariate autoregressive model (MVAR) to the sources and deriving a directed transfer function (DTF) from the resultant transfer function in the frequency domain [45]. For a multivariate k sourced process *X(t)* = (*X*_1_*(t),X*_2_*(t),….,X_k_(t)*), the model takes the form:

(1) $$X\left(t\right)=\overset{p}{\underset{j=1}{\sum }}A\left(j\right)X\left(t-j\right)+E\left(t\right)$$

where *E(t)* are vectors of size *k* and the coefficients *A* are *k* × *k* sized matrices. This equation can be transformed to describe relations in the frequency domain. After changing the sign of *A* and applying Z transform, Equations (2)–(4) can be derived:

(2) $$E\left(f\right)=A\left(f\right)X\left(f\right)\;$$

(3) $$X\left(f\right)=A^{-1}\left(f\right)E\left(f\right)=H\left(f\right)E\left(f\right)\;$$

(4) $$H\left(f\right)=\sum_{m=0}^{p}{\left(A\left(m\right)exp\left(-2\pi imf\;\Delta t\right)\right)}^{-1}\;$$

The model can be considered as a linear filter with white noise *E(f)* on its input and the signals *X(f)* on its output. The matrix of filter coefficients *H(f)* is the transfer matrix of the system. It contains information about all relations between source signals in the given set including the phase relations between signals. Based on the properties of the transfer matrix in MVAR, DTF is calculated according to Equation (5) [46]. The DTF describes the causal influence of source *j* on source *i* at frequency *f*. The above equation defines a normalized version of the DTF, which takes values from 0 to 1 producing a ratio between the inflow from source *j* to source *i* to all the inflows to source *i*. The DTF shows not only direct but also cascade flows.

(5) $$DTF_{j\to i}^{2}\left(f\right)=\frac{{\left|H_{i,j}\left(f\right)\right|}^{2}}{\sum_{m=1}^{k}{\left|H_{im}\left(f\right)\right|}^{2}}\;$$

For each participant, the order of the MVAR model was evaluated using the Akaike information criterion [13], implemented in MVGC multivariate Granger causality toolbox in MATLAB [14]. The model order was set to 15, as this was the most frequently occurring order over all participants. This was followed by obtaining the DTF spectrum, implemented in Fieldtrip. The DTF was averaged in theta (4–7 Hz), alpha (8–12 Hz), lower beta (13–20), and higher beta (21–30 Hz) bands. This resulted in 272 directed connections for each participant in each frequency band.

### 2.5. Statistics

Generalised linear models (GLM) were used to compare connectivity between groups. These models generalize regression analyses, integral to parametric ANOVA type tests, to discrete or continuous non-normal data [47]. This is facilitated via the use of a link function that transforms the response space into a modeling space, where the usual linear regression can be performed. 

The GLMs were implemented in RStudio (R version 4.2.0) using the “stats” package. For each of the 272 connections, a gamma family GLM with its canonical inverse link function was fitted with the group, frequency, and “group * frequency” interaction as fixed-effect predictors. Type III ANOVA-type *p*-Values were obtained and a correction for multiple comparisons was performed using the false discovery rate method [48]. The gamma family GLM was chosen as the connectivity distribution was positively skewed [49]. The model residuals were assessed for normality using the Shapiro–Wilk test (*p* < 0.05) and visual assessment of QQ plots. In case of a significant main effect of the group at a level of *p* < 0.05, post-hoc tests were conducted with Tukey’s method for correction of multiple comparisons. 

## 3. Results

### 3.1. Participant Details

The follow-up session revealed that among the twenty people who did not have CNP at the time of recording, ten developed CNP. The leg MI data was missing for two participants in the PdP group and one participant in the PwP group. The relevant demographics for all other participants are shown in Table 1. According to the American Spinal cord injury (ASIA) impairment scale, 60% of participants in PnP, 75% in PdP, and 50% in the PwP group had a complete injury, whereas others had an incomplete injury. Further, all participants in PnP, 75% in PdP, and 70% in the PwP group were paraplegics, whereas others were tetraplegics. All participants with pain reported pain larger than four on a visual numeric scale (range 0–10) and had below-level CNP in addition to at-level in some. The CNP-related demographics are presented in Appendix A Table A1.

### 3.2. Results of Statistical Analysis

**Figure 4:**
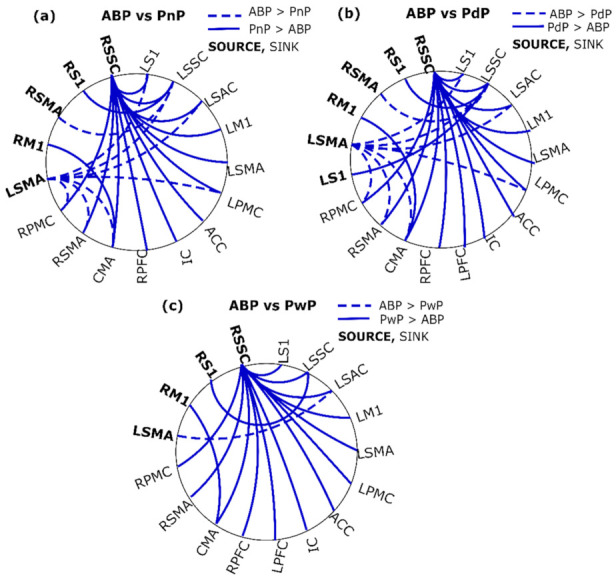
Significant differences in connectivity between able-bodied participants (ABP) and SCI groups: (**a**) PnP, (**b**) PdP, and (**c**) PwP. The SCI groups PwP, PnP, and PdP represent SCI participants with CNP at the time of recording, those who did not have CNP at the time of recording and remained CNP-free, and those who did not have CNP at the time of recording but developed CNP later. The solid lines indicate that the second group has greater connectivity than the first group, whereas the dashed lines indicate the opposite. Source areas are marked in bold whereas sink areas are non-bold.

**Figure 5:**
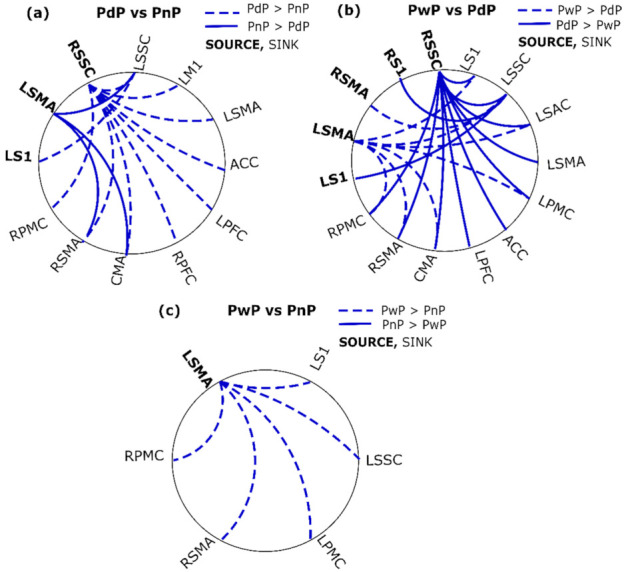
Significant differences in connectivity between SCI groups: (**a**) PdP vs. PnP, (**b**) PwP vs. PdP, and (**c**) PwP vs. PnP. PwP, PnP, and PdP represent SCI participants with CNP at the time of recording, those who did not have CNP at the time of recording and remained CNP-free, and those who did not have CNP at the time of recording but developed CNP later. The solid lines indicate that the second group has greater connectivity than the first group, whereas the dashed lines indicate the opposite. Source areas are marked in bold whereas sink areas are non-bold.

In total, 121 of the 272 model residuals were normally distributed according to the Shapiro–Wilks test. For the rest, the QQ plots indicated slight deviations from normality. The connections showing a significant difference are shown in Appendix A Table A2 along with *p*-values for the main effect of the group and mean and confidence intervals for each group. The results of the post-hoc analyses are presented in Figure 4 and Figure 5 for the differences between ABP and SCI groups, and differences within SCI groups, respectively.

**Figure A1:**
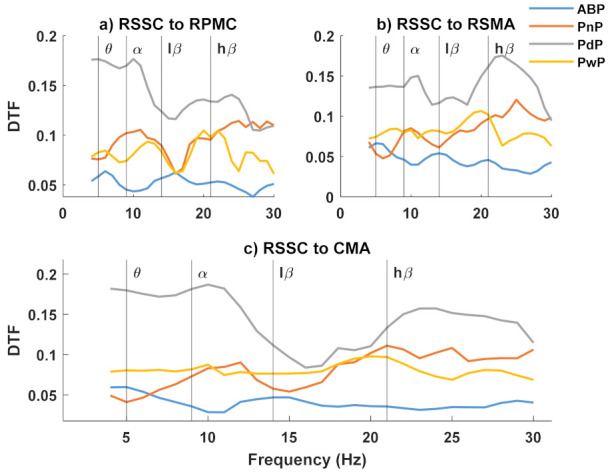
DTF spectrum for the connectivity from the right secondary somatosensory cortex (RSSC) to motor regions. (**a**) Right premotor cortex (RPMC), (**b**) right supplementary motor area (RSMA), and (**c**) cingulate motor area (CMA). θ, α, Iβ, and hβ represent theta, alpha, lower beta, and higher beta bands.

**Figure A2:**
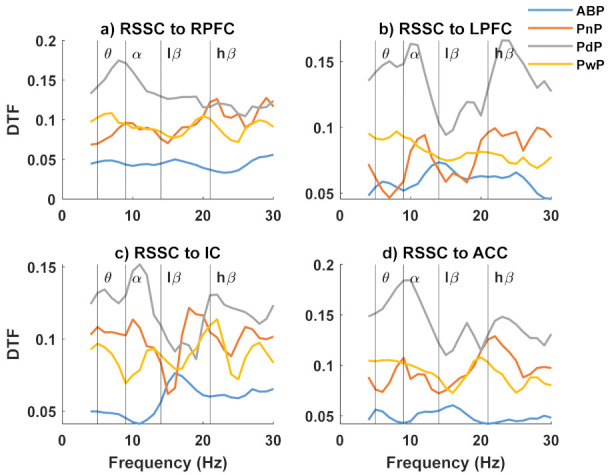
DTF spectrum for the connectivity from right secondary somatosensory cortex (RSSC) to regions of the pain matrix. (**a**) Right prefrontal cortex (RPFC), (**b**) left prefrontal cortex (LPFC), (**c**) insular cortex (IC), and (**d**) anterior cingulate cortex (ACC). θ, α, Iβ, and hβ represent theta, alpha, lower beta, and higher beta bands.

**Figure A3:**
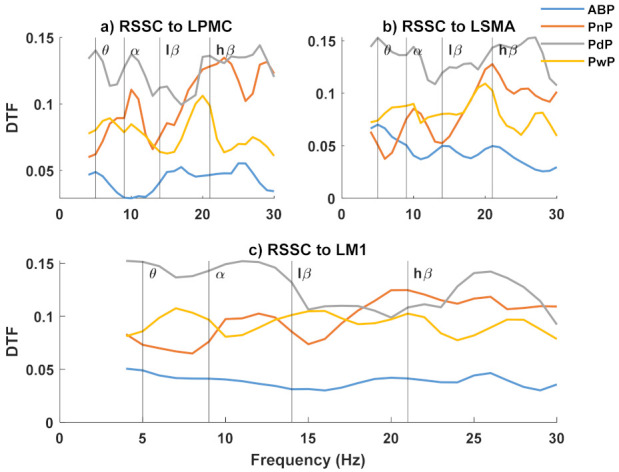
DTF spectrum for the connectivity from right secondary somatosensory cortex (RSSC) to motor regions. (**a**) Left premotor cortex (LPMC), (**b**) left supplementary motor area (LSMA), and (**c**) left primary motor cortex (LM1). θ, α, Iβ, and hβ represent theta, alpha, lower beta, and higher beta bands.

**Figure A4:**
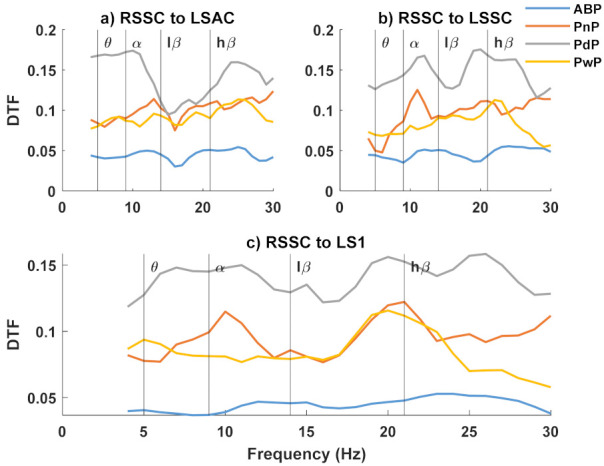
DTF spectrum for the connectivity from right secondary somatosensory cortex (RSSC) to sensory regions. (**a**) Left somatosensory association cortex (LSAC), (**b**) left secondary somatosensory cortex (LSSC), and (**c**) left primary somatosensory cortex (LS1). θ, α, Iβ, and hβ represent theta, alpha, lower beta, and higher beta bands.

**Figure A5:**
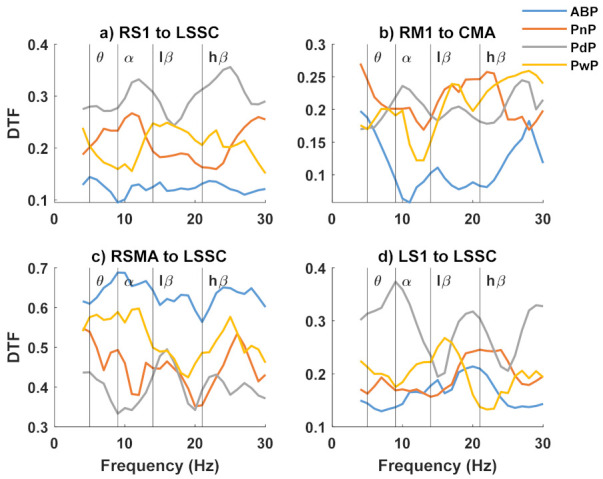
DTF spectrum for the connectivity from (**a**) right primary somatosensory cortex (RS1) to left secondary somatosensory cortex (LSSC), (**b**) right primary motor cortex (RM1) to cingulate motor area (CMA), (**c**) right supplementary motor area (RSMA) to LSSC, and (**d**) left primary somatosensory cortex (LS1) to LSSC. θ, α, Iβ, and hβ represent theta, alpha, lower beta, and higher beta bands.

**Figure A6:**
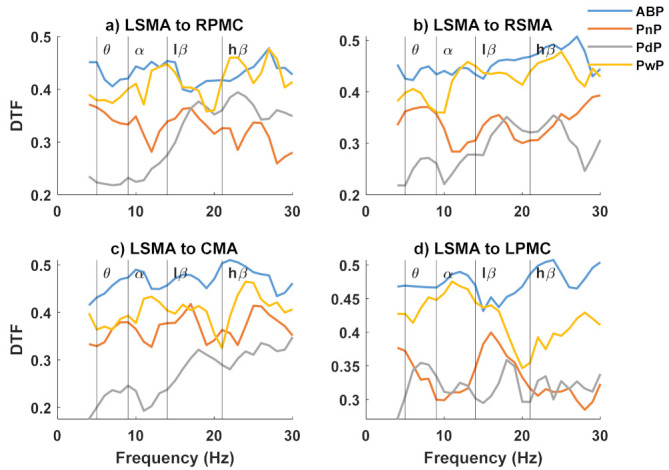
DTF spectrum for the connectivity from left supplementary motor area (LSMA) to motor regions. (**a**) Right premotor cortex (RPMC), (**b**) right supplementary motor area (RSMA), (**c**) cingulate motor area (CMA), and (**d**) left premotor cortex (LPMC). θ, α, Iβ, and hβ represent theta, alpha, lower beta, and higher beta bands.

**Figure A7:**
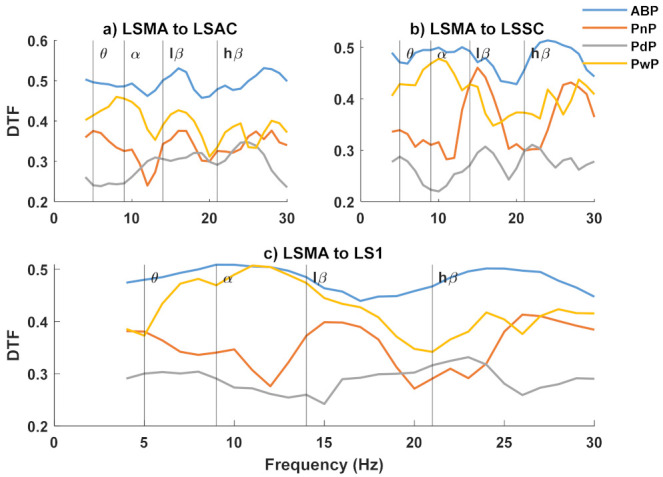
DTF spectrum for the connectivity from left supplementary motor area (LSMA) to sensory regions. (**a**) Left somatosensory association cortex (LSAC), (**b**) left secondary somatosensory cortex (LSSC), and (**c**) left primary somatosensory cortex (LS1). θ, α, Iβ, and hβ represent theta, alpha, lower beta, and higher beta bands.

No interactions between group and frequency were found, implying that the differences between groups are independent of the frequency band. The DTF spectrum for the connections showing a significant difference between any two groups is shown in Appendix A Figure A1, Figure A2, Figure A3, Figure A4, Figure A5, Figure A6 and Figure A7 for reference. 

### 3.3. Differences between ABP and SCI 

The connections showing a significant difference between ABP and SCI groups are presented in Figure 4. The ABP showed the most contrast to the PdP group, followed by PnP and PwP. These differences were governed mostly by connections from the RSSC and LSMA. Among sensory areas, all SCI groups showed increased outflow from the RSSC to left sensory areas, pain areas, and motor areas of both sides, except the RM1. The RS1 showed the same trend but only for its connection to LSSC. 

Among the motor areas, SCI groups PdP and PnP showed decreased outflow from the LSMA to the right motor areas and left sensory areas. PwP showed decreased outflow only from the LSMA to the LSAC. All SCI groups showed increased outflow from the RM1 to the CMA.

### 3.4. Differences between SCI Groups

The connections showing a significant difference within SCI groups are presented in Figure 5. The most differences were found between PdP and PwP, whereas PnP and PwP showed the fewest differences. The connections from RSSC were different between PdP and PwP as well as PdP and PnP, whereas PdP showed more outflow to the right motor, pain (except IC), left motor, and left sensory areas. The connectivity from the LSMA was significantly different between the three groups such that PwP > PnP > PdP. For the difference between PdP and PnP, the sinks were the RSMA, CMA, and LSSC. For the difference between PwP and PnP, the sinks excluded the CMA and additionally included the RPMC, LPMC, and LS1. For the difference between PwP and PdP, the LSAC was further involved in addition to the areas mentioned before. 

## 4. Discussion

We analysed how SCI and different phases of CNP affect connectivity during MI of the legs. The difference between able-bodied and SCI groups was larger than the differences within SCI groups: PwP (existing CNP), PnP (no CNP at the time of recording as well as on follow-up), and PdP (no CNP at the time of recording but developed CNP later). Therefore, it appears that paralysis affected connectivity more than CNP in the subacute phase. The differences between groups were independent of the frequency band. Although this has been demonstrated before when comparing effective connectivity between SCI and able-bodied people [24], it is possible that the frequency-specific differences were not large enough in the subacute phase of the injury. Most studies in the past have focused on chronic SCI whereas this study focused on subacute SCI. The first objective of the study was to differentiate between the effects of CNP and the effects of SCI. Based on our findings, subacute SCI seems to be associated with decreases in connectivity from the motor preparation region supplementary motor area and increases in connectivity to and from the secondary somatosensory cortex. CNP appears to reverse SCI-induced effective connectivity changes. 

MI recruits premotor regions such as the bilateral supplementary motor area (SMA) and premotor cortex which are involved in motor preparation [50]. Although we did not find changes in outflows from the premotor cortex between groups, outflows from the SMA presented differences across all frequency bands, being greater for ABP compared to PdP and PnP. In able-bodied volunteers, Alkinoos et al. showed a high output information exchange between the SMAs of both hemispheres as well as between the SMA and primary motor regions and attributed it to a regulative role of SMA during leg movement planning [51]. In a study by Fallani et al., the chronic SCI participants showed a remarkable outflow from SMA areas whereas the able-bodied participants showed an outflow from the foot primary motor cortex area in the beta frequency band during attempted movement of the right foot [25]. Hou et al. showed that resting-state functional connectivity decreases between bilateral SMAs and between the right M1 and right SMA are associated with poor recovery whereas the opposite is associated with good recovery [20]. Therefore, even though previous studies have identified SMA as an important outflow hub for SCI, our findings indicate that the strength of this outflow is still greater in able-bodied people. 

Contrary to the results at the SMA, all SCI groups showed greater outflow from the right primary motor cortex to the cingulate motor area compared to the ABP group. Whereas some studies have reported MI to be predominantly associated with cortical regions involved in the planning and preparation of movements, others point toward the participation of the primary motor cortex, albeit in a limited number of participants [52]. Previous studies have reported increases in primary motor cortex connectivity to other areas in both the subacute and chronic phases of SCI but in the resting state [19,21]. It might be possible that post-SCI, instead of premotor regions, primary and secondary sensorimotor areas get involved, as a form of motor adaptation [53]. Among all motor areas, only the cingulate motor area demonstrated increased inflow; it has been shown to be an important hub in both able-bodied and SCI motor networks [24,26,27] with the researchers attributing it to the increased effort required by SCI participants to perform a motor task of the paralysed limb [54,55]. 

The strength of outflows from the right secondary somatosensory cortex (SSC) and primary somatosensory cortex was greater for all SCI groups. We also found that sensory areas of the left hemisphere acted as sinks for the SMA, where decreased inflows were found. Even though the parietal cortex has been shown to be active during MI across many studies [56], the increased connectivity in subacute SCI compared to ABP can also be explained by posterior shifts in both upper limb and lower limb motor activation observed immediately post-SCI, which relocate anteriorly as recovery progresses [14]. Sensory areas project to the corticospinal tract and thus can compensate for the loss of primary motor cortex axons [57]. Even in chronic complete SCI, a larger involvement of the parietal cortex around MI onset has been shown [23]. The inflow of information from the cingulate motor area and SMA to the superior parietal cortex (which contains the somatosensory association cortex) was found to be a unique interaction present in the MI network for the chronic SCI cohort [26]. Cramer et al. demonstrated that although both SCI and controls showed an activation increase in the primary motor cortex during right foot MI, only the SCI group showed an activation increase in the primary somatosensory cortex [58]. Despitepeople with complete SCI lacking afferent inputs to the somatosensory cortex, the posterior shifts are still prevalent and rather permanent, possibly due to a lack of anterior shifts later, associated with lack of recovery [14,58]. Even though primary and secondary somatosensory cortices are part of the pain matrix [44], and increased sensory activation has been suggested owing to CNP [59], it can be ruled out here because the changes were seen across all SCI groups. 

The inflow to all pain areas from the right SSC was increased in SCI groups, irrespective of the CNP status. On one hand, it is likely that the MI period was not long enough to evoke CNP in the PwP group unlike previous studies [15,16]. On the other hand, all areas of the pain matrix [44] have been found to be associated with MI, such as the bilateral inferior frontal gyri and anterior insula [56], prefrontal cortex [60,61], and anterior cingulate cortex [54,55]. The anterior cingulate cortex plays a role in the affective or emotional component of pain, thus we would have expected increased connectivity only in the PwP group but this was not the case [62]. Moreover, PwP showed the fewest differences from ABP. Our research group has demonstrated an overactive sensorimotor cortex in chronic SCI participants with CNP [9,63]. It might be possible that an overactive sensorimotor cortex is also present in the subacute stage and reversed the effect of SCI on motor areas. This overactivation might be a cause, or an effect of hyperconnectivity between somatomotor components demonstrated previously in chronic CNP [16]. Jutzeler et al. showed that people with chronic SCI and CNP are more similar in activation to able-bodied controls than those that are CNP free, and demonstrate less cortical reorganization [64]. The authors interpreted that painful sensory input arising from deafferented areas of the body might have maintained cortical representation, or inversely, a lack of cortical reorganization may have caused CNP. Our findings are reflective of a similar trend in the subacute phase.

The second objective of the study was to find predictors of CNP in the connectivity patterns. The differences between different SCI groups involved the same areas as those governing differences between ABP and SCI groups, namely, the right SSC and left SMA. The outflow from the left SMA to right motor areas and left sensory areas were different between the three groups such that it was highest for PwP, followed by PnP, and the lowest for PdP. In the context of ABP vs SCI findings, where we hypothesized that hyperconnectivity owing to CNP may have reversed the effect of SCI, the PwP group having the largest connectivity among SCI groups for SMA outflows is plausible. With this logic, we would have expected the largest connectivity of the PwP group even for the right SSC outflows as it is part of the pain matrix. In fact, Wrigley et. al. showed that somatosensory reorganization was correlated with ongoing pain intensity in chronic complete SCI [65]. However, in this study, instead of PwP who had ongoing pain, the PdP group, who developed CNP later, showed the largest outflows from the right SSC. It has been proposed that central sensitization in nociceptive pathways and adaptive plasticity of motor learning share common mechanisms and compete with each other [66]. It could be that hyperconnectivity from the right SSC might serve an adaptive purpose initially to support efferent activation, however, in the long run, might contribute to CNP symptoms (maladaptive plasticity) if not reversed. 

The findings of this study might be useful to guide brain-targeted interventions. Areas such as the SMA [67], premotor cortex [68], and primary motor cortex [69] have been the target for upregulation in neurofeedback studies, consequently increasing their functional connectivity to other brain regions. Neurofeedback interventions also target connectivity between motor regions directly [70]. As decreased SMA connectivity is associated with poor recovery, the SMA could be the most likely therapeutic target for neurofeedback-based SCI treatments to enhance motor recovery. Somatosensory stimulation has been shown to augment functional recovery [71], increase cortical excitability [72], and influence reorganization positively [73]. The results of this study support somatosensory stimulation because as the somatosensory areas get more connected to the corticospinal tract via an increase in afferent (sensory) inputs, the hyperconnectivity of those areas can decrease for efferent (motor) output (a compensatory mechanism) and gradually contribute to anterior shifts (original representation) in activation. Moreover, CNP status should be monitored through these interventions as changes in sensory connectivity might be related to both adaptive and maladaptive plasticity.

In a previous work on this dataset, Vuckovic et al. (2018)compared age between groups using ANOVA and found no statistical differences [34]. However, SCI groups with pain had several participants quite older than the able-bodied group. Ageing has been associated with enhanced connectivity in the core motor network (i.e., premotor cortex and primary motor cortex) [74,75,76,77] as well between prefrontal and motor areas corresponding to upper limb movement [78]. For the lower limb MI, similar connectivity studies are scarce however activation studies point towards increased activity of premotor, prefrontal, and somatosensory cortices [79,80]. Among all findings, the increased connectivity from the somatosensory cortices to prefrontal and premotor areas, for the SCI group, may have been influenced by age. However, at the same time, there was a decreased connectivity from the premotor supplementary motor area in all SCI groups compared to the able-bodied group. Nevertheless, the influence would be to a lower degree as ageing studies typically have an average age of 60, whereas the average age in this study was less than 50 for the SCI groups.

There are several limitations of this study. The first is a small sample size, although it is in line with similar studies on SCI connectivity [24,27,81]. The second limitation is that the SCI participants are of mixed characteristics—paraplegic and tetraplegic, both complete and incomplete, therefore potentially confounding the analysis. We tried to minimize this effect by analysing MI of legs only as it was affected in all SCI participants and the region where most participants of both CNP groups experienced CNP. 

The next limitation of this study is the use of source reconstruction to derive cortical signals from scalp signals, as it is an ill-defined problem, and several factors affect the accuracy of derivatives such as the use of standard MRI as opposed to subject-specific MRI and how well the actual sources comply with the assumption of source model [82]. However, eLORETA seemed suitable for the analysis as deeper sources were included in the regions of interest and it lowers localisation error for deeper sources [42]. It has also been shown to be more accurate than its more popular counterpart sLORETA [83]. Finally, surrogate distributions were not used to threshold the original connectivity distribution, a recommended method to avoid spurious connectivity due to noise or volume conduction [84]. However, the connectivity method used is insensitive to volume conduction and very robust with respect to noise [45].

Overall, the results of this study encourage future connectivity research on SCI and CNP with large sample sizes to enable analysis considering the various confounders such as age, level of injury, and completeness of injury into account. Future studies should be longitudinal andrecord motor recovery along with CNP status to address the interaction between their effects on cortical connectivity. Further, structural MRIs could be recorded for each participant to increase the accuracy of derived EEG sources. 

## 5. Conclusions

Effective connectivity during MI of a painful part of the body is influenced more by SCI than CNP. CNP seems to reduce the effects of motor connectivity changes owing to SCI. The cortical and subcortical areas typically associated with CNP but also associated with MI, do not show a differential connectivity pattern for SCI-induced CNP. Changes in sensory connectivity might reflect adaptive as well as maladaptive plasticity. 

## Figures and Tables

**Table 1:** Demographics of the three groups of SCI participants. Lev and Com correspond to ASIA impairment scale level, and completeness of injury respectively [35]. ABP: able-bodied participants; PnP: SCI participants who had no pain at the time of EEG recording and did not develop pain later; PdP: SCI participants who had no pain at the time of EEG recording but developed pain later; PwP: SCI: participants who had pain at the time of EEG recording. Medications PG, GP, and TR refer to pregabalin, gabapentin, and tramadol respectively. A: complete loss of sensory and motor function; B: complete loss of motor functions and some sensory function spared, C: and D: incomplete loss of both sensory and motor function, motor impairment being larger in group C. Data are presented as mean (M) and standard deviation (SD).

Group	No	1	2	3	4	5	6	7	8	9	10	M (SD)
ABP	Age	37	32	36	34	32	27	45	34	49	27	35 (7)
PnP	Age	51	22	47	41	59	43	24	38	62	34	42 (13)
	Lev	T7/T10	L1	T11	T12	T6	T6/T7	L1	L1	T3/T5	T6	-
	Com	D	B	D	A	A	B	A	A	A	A	-
	Weeks with SCI	12	12	7	4	12	21	7	4	10	10	10 (5)
PdP	Age	70	49	19	69	32	46	49	32	-	-	46 (18)
	Lev	T7/T8	T12	C5/C6	L2	T3	T5	T6	C3	-	-	-
	Com	D	A	A	B	A	A	A	A	-	-	-
	Weeks with SCI	9	6	12	6	24	6	4	6	-	-	9 (6)
PwP	Age	33	59	27	32	30	59	29	37	49	75	43 (16)
	Lev	T12	T7/T8	C5/C6	T3	T10	T8	C3	T6	C4	T6	-
	Com	B	A	A	A	A	C	D	B	A	C	-
	Weeks with SCI	20	12	17	24	12	26	6	28	6	6	16 (9)

## Data Availability

The data will be available on request.

## Abbreviations

SCI—spinal cord injury; CNP—central neuropathic pain; EEG—electroencephalogram; fMRI—functional magnetic resonance imaging; MI—motor imagery; ABP—able bodied participants; PwP—SCI participants who had CNP at the time of recording; PnP—SCI participants who did not develop CNP; PdP—SCI participants who developed CNP; ICA-independent components analysis; MRI—magnetic resonance imaging; eLORETA—exact low resolution electromagnetic tomography; RPMC—right premotor cortex; LPMC—left premotor cortex; RSMA—right supplementary motor area; LSMA—left supplementary motor area; RM1—right primary motor cortex; LM1—left primary motor cortex; RSAC—right somatosensory association cortex; LSAC—left somatosensory association cortex; RSSC—right secondary somatosensory cortex; LSSC-left secondary somatosensory cortex; RS1—right primary somatosensory cortex; LS1-left primary somatosensory cortex; CMA—cingulate motor area; RPFC—right prefrontal cortex; LPFC—left prefrontal cortex; IC—insular cortex; ACC—anterior cingulate cortex; MVAR—multivariate autoregressive model; DTF—directed transfer function; GLM—generalized linear model; ASIA—American spinal injury association; PG—pregabalin; GP—gabapentin; TR—tramadol; M-mean; SD—standard deviation; CI—confidence intervals; VNS—visual numeric scale.

## Appendix A

**Table A1:** Demographics of groups with existing pain (PwP) and future pain (PdP). VNS refers to a visual numeric scale, where participants were asked to select their pain level on a 10-point scale where 0 represents no pain and 10 represents the worst pain imaginable.

Group	No	1	2	3	4	5	6	7	8	9	10	M (SD)
PdP	Weeks with pain (post-recording)	6	10	4	4	8	7	2	4	-	-	6 (3)
	Location	Feet	At and below level	Hands	Left leg	At and below level	At and below level	At and below level	Hands	-	-	-
	Pain VNS	4	2	6	1	4	4	6	2	-	-	4 (2)
PwP	Weeks Before pain	20	12	15	6	12	26	6	28	6	6	14 (8)
	Location	At and below level	At and below level	Hands and Buttock	At and below level	Legs and feet	At level and feet	Right hand	Right leg	Hands	At and below level	-
	Pain VNS	9	6	5	5	7	5	6	8	7	7	6 (1)
	Meds	PG	GP	TR	PG	-	-	-	PG	GP	GP	-

**Table A2:** GLM *p*-Values of the main effect of group in connections showing a significant difference. The mean (M) and confidence interval (CI) of the connectivity values are stated for those groups. ABP: able-bodied participants; PnP: SCI participants who had no pain at the time of EEG recording and did not develop pain later; PdP: SCI participants who had no pain at the time of EEG recording but developed pain later; PwP: SCI: participants who had pain at the time of EEG recording.

S no.	Connection	ABP M [95% CI]	PnP M [95% CI]	PdP M [95% CI]	PwP M [95% CI]	Main *p*-Value	FDR Adjusted
1	RSSC to RPMC	0.05 [0.04 0.06]	0.09 [0.08 0.11]	0.14 [0.12 0.18]	0.08 [0.07 0.10]	0.0002	0.0072
2	RSSC to RSMA	0.05 [0.04 0.06]	0.07 [0.06 0.09]	0.14 [0.11 0.18]	0.08 [0.07 0.10]	0.0027	0.0348
3	RSSC to CMA	0.04 [0.03 0.05]	0.07 [0.06 0.09]	0.14 [0.12 0.19]	0.08 [0.07 0.10]	2.61 × 10^−6^	0.0003
4	RSSC to RPFC	0.04 [0.04 0.06]	0.09 [0.07 0.11]	0.14 [0.11 0.18]	0.09 [0.08 0.12]	0.0007	0.0129
5	RSSC to LPFC	0.06 [0.05 0.07]	0.07 [0.06 0.09]	0.14 [0.11 0.17]	0.08 [0.07 0.10]	0.0015	0.0227
6	RSSC to I	0.05 [0.04 0.07]	0.10 [0.08 0.13]	0.12 [0.10 0.15]	0.09 [0.07 0.11]	0.001	0.0171
7	RSSC to ACC	0.05 [0.04 0.06]	0.09 [0.08 0.11]	0.14 [0.12 0.19]	0.09 [0.08 0.12]	0.0004	0.0109
8	RSSC to LPMC	0.04 [0.03 0.05]	0.09 [0.08 0.11]	0.12 [0.10 0.16]	0.08 [0.07 0.10]	1.15 × 10^−5^	0.001
9	RSSC to LSMA	0.05 [0.04 0.06]	0.07 [0.06 0.09]	0.13 [0.11 0.17]	0.08 [0.07 0.10]	0.0028	0.0351
10	RSSC to LM1	0.04 [0.03 0.05]	0.09 [0.08 0.11]	0.13 [0.11 0.16]	0.09 [0.08 0.11]	4.24 × 10^−5^	0.0019
11	RSSC to LSAC	0.04 [0.04 0.06]	0.10 [0.08 0.12]	0.14 [0.12 0.19]	0.09 [0.07 0.12]	0.0013	0.0213
12	RSSC to LSSC	0.05 [0.04 0.06]	0.09 [0.07 0.11]	0.14 [0.12 0.18]	0.08 [0.07 0.10]	0.0002	0.0061
13	RSSC to LS1	0.04 [0.04 0.06]	0.09 [0.08 0.12]	0.14 [0.11 0.19]	0.08 [0.07 0.11]	0.0021	0.021
14	RS1 to LSSC	0.12 [0.10 0.15]	0.21 [0.18 0.26]	0.29 [0.24 0.37]	0.20 [0.17 0.25]	0.0037	0.0425
15	RM1 to CMA	0.11 [0.09 0.13]	0.21 [0.18 0.26]	0.20 [0.17 0.24]	0.20 [0.17 0.23]	0.0006	0.0129
16	RSMA to LSSC	0.64 [0.57 0.72]	0.45 [0.41 0.51]	0.40 [0.35 0.45]	0.53 [0.48 0.60]	0.0006	0.0129
17	LSMA to RPMC	0.43 [0.39 0.48]	0.33 [0.30 0.37]	0.27 [0.25 0.31]	0.41 [0.37 0.45]	0.0003	0.0079
18	LSMA to RSMA	0.45 [0.41 0.50]	0.34 [0.31 0.38]	0.27 [0.25 0.31]	0.42 [0.38 0.46]	0.0023	0.031
19	LSMA to CMA	0.46 [0.42 0.51]	0.36 [0.33 0.40]	0.25 [0.22 0.28]	0.40 [0.36 0.44]	1.75 × 10^−5^	0.0012
20	LSMA to LPMC	0.47 [0.43 0.52]	0.33 [0.30 0.37]	0.32 [0.29 0.36]	0.43 [0.39 0.47]	0.001	0.0171
21	LSMA to LSAC	0.49 [0.45 0.55]	0.34 [0.31 0.37]	0.28 [0.25 0.31]	0.40 [0.37 0.44]	6.52 × 10^−5^	0.0025
22	LSMA to LSSC	0.48 [0.44 0.53]	0.34 [0.31 0.38]	0.27 [0.24 0.30]	0.42 [0.38 0.46]	3.68 × 10^−8^	1.00 × 10^−5^
23	LSMA to LS1	0.48 [0.44 0.54]	0.35 [0.32 0.39]	0.29 [0.26 0.32]	0.43 [0.39 0.48]	2.21 × 10^−5^	0.0012
24	LS1 to LSSC	0.16 [0.13 0.19]	0.19 [0.16 0.22]	0.29 [0.25 0.36]	0.20 [0.17 0.24]	0.0035	0.0421
